# The effect of pregnancy vitamin D supplementation on maternal blood pressure: real-world data analysis within the MAVIDOS randomised placebo-controlled trial

**DOI:** 10.1007/s00404-025-07958-z

**Published:** 2025-01-30

**Authors:** Natasha L. Citeroni-Clark, Stefania D’Angelo, Sarah R. Crozier, Alexandra Kermack, Keith M. Godfrey, Cyrus Cooper, Nicholas C. Harvey, Rebecca J. Moon

**Affiliations:** 1 MRC Lifecourse Epidemiology Centre, University of Southampton, Southampton General Hospital, Tremona Road, Southampton, SO16 6YD UK; 2 MRC Lifecourse Epidemiology Centre, MRC Versus Arthritis Centre for Musculoskeletal Health and Work, University of Southampton, Southampton, UK; 3 NIHR Applied Research Collaboration Wessex, Southampton Science Park, Innovation Centre, 2 Venture Road, Chilworth, Southampton, SO16 7NP UK; 4 NIHR Southampton Biomedical Research Centre, University of Southampton and University Hospital Southampton NHS Foundation Trust, Southampton General Hospital, Tremona Road, Southampton, SO16 6YD UK; 5 Department of Obstetrics and Gynaecology, University Hospital Southampton NHS Foundation Trust, Princess Anne Hospital, Coxford Road, Southampton, SO16 5YA UK; 6 School of Medicine, Faculty of Health and Medical Sciences, University of Surrey, Guildford, UK; 7 NIHR Biomedical Research Centre, University of Oxford, Oxford, UK; 8 Paediatric Endocrinology, Southampton Children’s Hospital, University Hospital Southampton NHS Foundation Trust, Southampton General Hospital, Tremona Road, Southampton, SO16 6YD UK

**Keywords:** Hypertensive disorders of pregnancy, Preeclampsia, Pregnancy-induced hypertension, Cholecalciferol

## Abstract

**Purpose:**

Observational studies have suggested negative associations between maternal 25-hydroxyvitamin D (25(OH)D) status and risk of hypertensive disorders of pregnancy [pregnancy-induced hypertension (PIH) and preeclampsia (PET)]. Data from intervention studies are limited. We hypothesised that vitamin D supplementation would lower maternal blood pressure (BP) during pregnancy and reduce the incidence of hypertensive disorders of pregnancy.

**Methods:**

The Maternal Vitamin D Osteoporosis Study (MAVIDOS) was a randomised placebo-controlled trial. Pregnant women with a baseline 25(OH)D of 25–100 nmol/l were randomized to either 1000 IU/day cholecalciferol or placebo from 14 to 17 weeks’ gestation until delivery. BP recordings documented during routine clinical pregnancy care were obtained from clinical records and grouped into gestational windows based on the schedule for routine antenatal care in the United Kingdom (23^+0^–24^+6^, 27^+0^–28^+6^, 33^+0^–35^+6^, 37^+0^–38^+6^, 39^+0^–40^+6^ and ≥ 41^+0^ weeks^+days^). Systolic and diastolic BP measurements in these gestational windows were compared between randomisation groups. Diagnoses of PIH or PET (in accordance with national guidelines) and the use of antihypertensive agents were also noted and compared between groups.

**Results:**

Data for 734 women (366 cholecalciferol, 368 placebo) were included. Maternal mean systolic and diastolic BP did not differ between the randomization groups at any of the gestations studied. The incidences of PIH (placebo 1.6%, cholecalciferol 3.6%, *p* = 0.10) and PET (placebo 3.3%, cholecalciferol 3.8%, *p* = 0.68) were similar between the two groups.

**Conclusions:**

Gestational vitamin D supplementation with 1000 IU/day from 14 to 17 weeks gestation did not lower maternal BP or reduce the incidences of PIH or PET in this trial.

**Supplementary Information:**

The online version contains supplementary material available at 10.1007/s00404-025-07958-z.

## What does this study add to the clinical work

**Table:** 

Observational data have suggested that a higher maternal serum 25-hydroxyvitamin D status in pregnancy may reduce the risk of hypertensive disorders of pregnancy. In this post-hoc analysis of a large randomized controlled trial, 1000 IU/day cholecalciferol from 14–17 weeks' gestation until delivery did not lower maternal blood pressure during pregnancy or reduce the incidence of pregnancy induced hypertension or preeclampsia, suggesting that moderate dose vitamin D supplementation from mid-pregnancy may not be an effective approach to address these clinically important outcomes.	

## Background

Hypertensive disorders of pregnancy, including pregnancy-induced hypertension (PIH) and pre-eclampsia (PET) affect around 5–10% and 2–5% of all pregnancies, respectively [1]. These conditions may have significant consequences for the mother, including maternal end-organ damage and death, and for the fetus, such as fetal growth restriction, preterm delivery and intrauterine death [2].

Several risk factors for hypertensive disorders of pregnancy have been identified, including a previous history or family history of PIH or PET, nulliparity, raised pre-pregnancy body mass index (BMI), multiple pregnancies, inter-pregnancy interval greater than 10 years, pre-existing renal disease and autoimmunity [3]. However, the exact pathophysiologies of PIH and PET are not fully understood; abnormal placental implantation, vascular endothelial damage, immune dysregulation, coagulopathy and genetic factors are proposed mechanisms [4]. Most recently, a potential role of the maternal cardiovascular system has also been proposed [5]. This is in part due to the known increased risk for women with underlying hypertensive and cardiorenal disorders, in addition to greater circulating antiangiogenic factors associated with abnormal vascular function observed in both PIH and PET [6–8].

An effect of maternal vitamin D status on the risk of PIH and PET has also been hypothesised due to the recognised role of vitamin D in immunological function and the possible impact of this on placental function. Indeed, in a rodent model of PET, CD4 + T cells and pro-inflammatory cytokines were increased compared to normal pregnancy, but these and mean arterial pressure were reduced by vitamin D supplementation [9]. Several observational studies have shown negative associations between serum 25-hydroxyvitamin D [25(OH)D] in pregnant women and the incidence of PET [10, 11], but this is not consistent across all studies [12]. However, observational studies related to vitamin D are limited by confounding and reverse causality. Several randomised controlled trials (RCTs) have assessed the effect of vitamin D on PIH and/or PET incidence, but these studies are often underpowered or have recruited women at high risk of hypertensive disorders of pregnancy [13, 14]. While meta-analyses of these studies have suggested that vitamin D supplementation may be of benefit in reducing PET [13, 14], care should be taken in the interpretation of this data as several of the included studies had very high incidences of PET in the control group compared to general population data [13–15].

We hypothesised that vitamin D supplementation in pregnancy would lower maternal BP during pregnancy, and as a result would reduce the incidence of hypertensive disorders of pregnancy. We sought to examine this by integrating real-world BP data collected during pregnancy care with trial data within the MAVIDOS RCT of vitamin D supplementation in pregnancy [16, 17].

## Methods

MAVIDOS was a multi-centre double-blind randomised placebo-controlled trial of vitamin D supplementation in pregnancy. The primary outcome was offspring bone mass at birth. Full details of the study methodology and primary outcome have been previously published [17]. The trial was conducted in accordance with the Declaration of Helsinki guidelines and was approved by the Southampton and South-West Hampshire Research Ethics Committee. Full approval from the UK Medicines and Healthcare products Regulatory Agency (MHRA) was granted. All women gave written informed consent to participate in the pregnancy phase of the study. MAVIDOS was registered on the International Standard Randomised Controlled Trial registry, ISRCTN 82927713, and the European Clinical Trials Database, EudraCT 2007–001716–23 [18]. To increase the transparency in the design and conduct of this analysis, the research question and protocol were published in the Open Science Framework prior to data analysis [19].

In brief, women aged > 18 years with a singleton pregnancy attending one of three research centres in the United Kingdom (University Hospital Southampton NHS Foundation Trust, Oxford University Hospitals NHS Foundation Trust and Sheffield Hospitals NHS Trust) between 11 and 14 weeks’ gestation for dating scanning between 10th October 2008 and 11th February 2014 were invited to participate in the study. Owing to an ethical stipulation, only women with a baseline 25(OH)D measured on the local hospital platform [all three laboratories participate in the Vitamin D External Quality Assessment Scheme (DEQAS) vitamin D quality assurance system (http://www.deqas.org/)] between 25 and 100 nmol/l were allowed to participate. Women were randomized in a 1:1 ratio to either oral cholecalciferol 1000 IU/day or matched placebo started from 14 to 17 weeks' gestation and continued until delivery. All women received standard antenatal and intrapartum care delivered by health professionals blinded to the study allocation and were able to continue taking up to 400 IU/day vitamin D supplementation [17, 18].

### Maternal pregnancy assessments as part of the MAVIDOS RCT

Assessments of maternal lifestyle, health and nutrition by interviewer-led questionnaire and anthropometry were performed at randomization (14–17 weeks’) and 34 weeks’ gestation. Non-fasted venous blood samples were collected at these two visits. Serum was stored at − 80 °C. 25(OH)D concentration was assessed by chemiluminescence immunoassay (Liaison automated platform, Diasorin, Minnesota, USA). All samples were analysed in a single batch at Medical Research Council (MRC) Human Nutrition Research, Cambridge, UK. Within- and between-assay coefficients of variation were 4.1 and 6.1% [20].

### Maternal blood pressure

For women recruited from University Hospital Southampton NHS Foundation Trust and continuing in the study until delivery, maternal BP and urinalysis data collected during the pregnancy and recorded diagnoses of hypertension, PIH and PET, associated biochemistry results and anti-hypertensive use were extracted from clinical records.

Typically for nulliparous women in the UK, BP is measured at routine antenatal appointments occurring at pregnancy booking, 16 weeks’, 25 weeks’, 28 weeks’, 31 weeks’, 34 weeks’, 36 weeks’, 38 weeks’, 40 weeks’, 41 weeks’, and 42 weeks’ gestation; and for multiparous women, BP is assessed at booking, 16 weeks’, 28 weeks’, 34 weeks’, 36 weeks’, 38 weeks’, 41 weeks’, and 42 weeks’ gestation [21, 22]. Additional BP measurements may have been taken if women required additional monitoring or presented for non-routine clinical review. Only BP measurements prior to delivery were included in the analysis.

Gestation at each BP measurement was calculated using the infant’s date of birth and gestation at birth when compared to the calculated date of conception. Date of BP measurement was then used to establish gestation at measurement. BP measurements were grouped into gestational windows due to known variations in BP throughout pregnancy relating to peripheral vascular resistance [23]. The gestational windows were chosen to reflect the typical windows of BP assessment (as described above) as follows: baseline (< 12^+6 days^), 23^+0 days^–24^+6 days^, 27^+0 days^–28^+6 days^, 33^+0 days^–35^+6 days^, 37^+0 days^–38^+6 days^, 39^+0 days^–40^+6 days^ and ≥ 41^+days^. A gestational window including the 16 weeks’ gestation assessment was not included as some, but not all, women had been randomised to the study at this gestation.

All BP measurements were reviewed for outliers. A systolic BP documented as ≥ 140 or ≤ 80 mmHg or a diastolic measurement ≥ 90 or ≤ 50 mmHg was reviewed by a trained clinician in the trend of the other BP measurements and for the use of anti-hypertensive agents; those considered to be anomalous were removed. In instances where multiple BP measurements were recorded within the same gestational window, the latest gestation measurement was used.

For any systolic BP ≥ 140 mmHg or diastolic BP ≥ 90 mmHg, the clinical notes were reviewed to establish if anti-hypertensive medication was commenced. If anti-hypertensive medication was initiated by the clinical team, the last BP before starting medication was included. The participant was then censored from further data inclusion. Participants with pre-existing hypertension were included in analysis and censored if a change to their medication was required.

### Hypertensive disorders of pregnancy

A participant was considered to have PIH if they had 2 or more consecutive BP ≥ 140 mmHg systolic or ≥ 90 mmHg diastolic [22] or if they were started on antihypertensive treatment according to the clinical notes. The presence of PET was based on recognised diagnostic criteria of urine protein: creatinine ratio (PCR) ≥ 30 mg/mmol where available, or a clinical diagnosis with or without supporting biochemistry by the obstetric team managing the patient [22].

### Statistical methods

Data were analysed on an intention-to-treat basis. The distribution of continuous variables was assessed by visual inspection. Comparisons between randomisation groups and between those included and not included were made using t-tests, Mann–Whitney *U* tests and Chi-squared tests for normally distributed continuous, non-normally distributed continuous and categorical variables, respectively. Fisher’s exact test was used in case the number of observations was below 5. Gestation at the point when anti-hypertensive agents were started and at delivery was compared between groups using Mann–Whitney *U* test. BP comparison was undertaken separately for nulliparous and multiparous women due to the recognised differing risk of PIH/PET by parity [24]. Results are reported as mean (standard deviation [SD]), median (interquartile range [IQR]) and *n* (%) for normally distributed continuous, non-normally distributed continuous and categorical variables, respectively. Statistical analysis was performed using Stata 17.0 [25].

The MAVIDOS trial was originally powered to detect a difference in offspring bone mass [17, 18]. Nonetheless, a study including 360 women in each group and a background rate of PET of 3.5% (in the placebo group) would have 80% statistical power at the 5% level to detect a reduction in PET incidence of 2.9%.

## Results

**Fig. 1:**
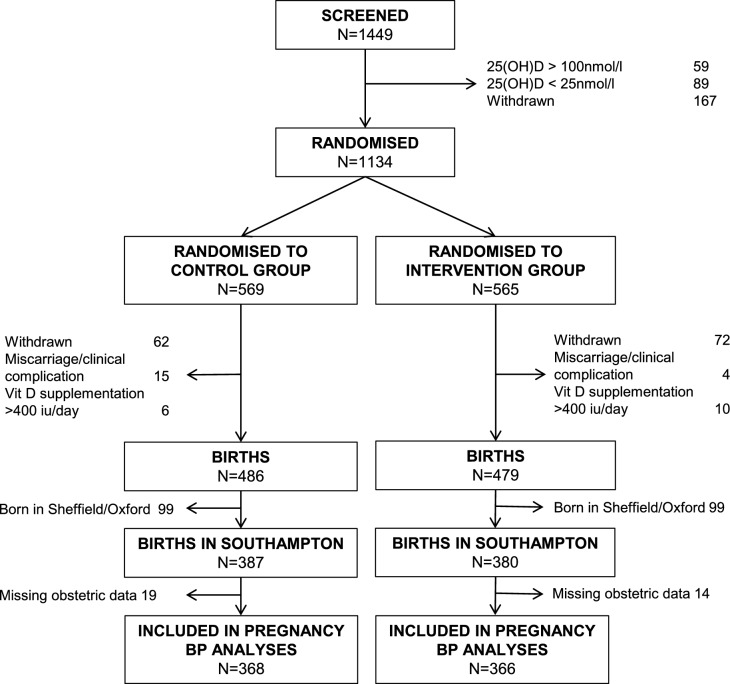
Consort diagram

1134 women were randomised in the MAVIDOS study, of whom 965 remained in the study until delivery. 767 (387 placebo, 380 cholecalciferol) study participants with a pregnancy resulting in a liveborn infant were recruited in Southampton; 33 of these were missing obstetric records. Thus 734 women were included in the analysis (Fig. 1). One participant included for analyses of hypertensive disorders of pregnancy prevalence, was not included in the BP analysis due to uncertainty on the use of antihypertensive therapy.

The women included in this analysis were of similar age at delivery, ethnicity, parity, smoking status, education, initial serum 25(OH)D status and systolic and diastolic BP at booking to women not included in this analysis (Online Resource Supplementary Table 1).

Baseline maternal characteristics between the two randomisation groups are shown in Table 1, and separately for nulliparous and multiparous women available in Online Resource Supplementary Tables 2 and 3, respectively. The two randomisation groups had similar serum 25(OH)D at randomisation (Table 1), but serum 25(OH)D was higher in the women randomised to cholecalciferol at 34 weeks’ gestation (placebo: 41.4 (SD 20.7) nmol/l; cholecalciferol 66.2 (SD 20.3) nmol/l, *p* < 0.001) as an anticipated consequence of the trial intervention.

**Table 1:** Baseline characteristics of women randomised to placebo or 1000 IU/day cholecalciferol at 14–17 weeks’ gestation until delivery

	Placebo (*n* = 368)		Cholecalciferol (*n* = 366)	
	*n*		*n*	
Age at delivery (years), mean (SD)	368	31.2 (5.1)	366	31.1 (5.1)
White ethnicity, *n* (%)	367	349 (95.1%)	363	347 (95.6%)
Nulliparous, *n* (%)	364	154 (42.3%)	365	156 (42.7%)
Height (cm), mean (SD)	364	166.0 (6.7)	365	165.5 (6.2)
Weight (kg), mean (SD)	368	73.3 (14.2)	366	70.9 (14.1)
BMI (kg/m^2^), mean (SD)	364	26.6 (5.0)	365	25.9 (4.9)
Smoking in early pregnancy, *n* (%)	366	32 (8.7%)	365	28 (7.7%)
Moderate/strenuous physical activity (hours/week), mean (SD)	284	0.95 (0.81)	287	0.89 (0.66)
Educated to A level or more, *n* (%)	363	271 (74.7%)	364	280 (76.9%)
Pre-existing hypertension, *n* (%)	368	4 (1.1%)	366	3 (0.8%)
Systolic BP at booking (mmHg), mean (SD)	310	110 (11)	305	109 (10)
Diastolic BP at booking (mmHg), mean (SD)	311	66 (9)	305	66 (8)
25(OH)D at randomisation (nmol/l), mean (SD)	360	44.4 (16.4)	357	45.6 (16.4)

**Fig. 2:**
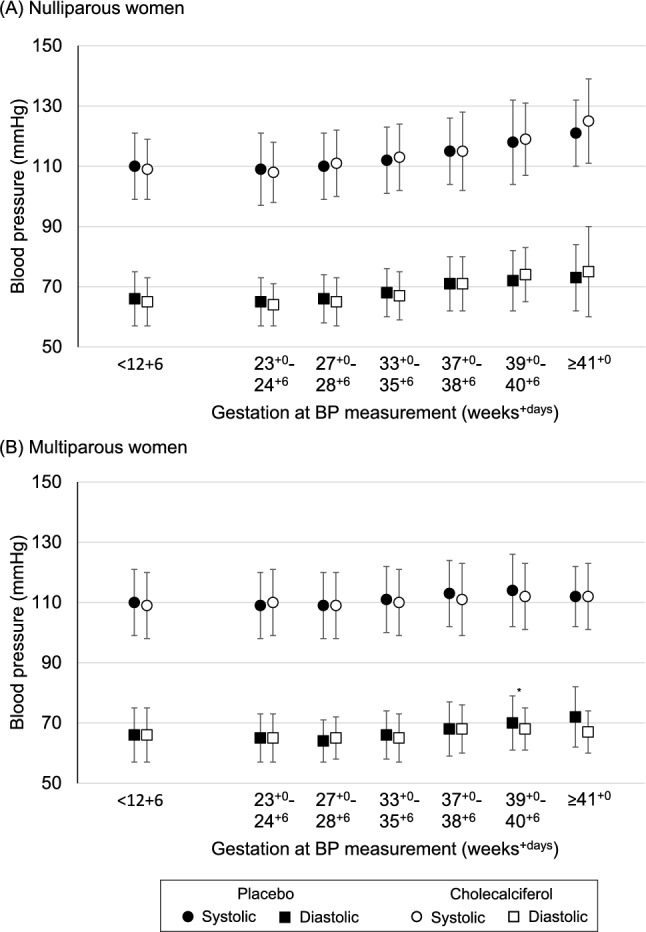
Systolic (shown by circles) and diastolic (shown by squares) blood pressure in women randomised to either placebo (black) or 1000 IU/day cholecalciferol (white) from 14–17 weeks gestation until delivery. Shown as mean (SD). *p = 0.05

There were no differences in maternal systolic or diastolic BP at any of the gestations considered by the randomisation group for nulliparous women (Fig. 2A and Online Resource Supplementary Table 4), multiparous women (Fig. 2(B) and Online Resource Supplementary Table 5), or for all women combined (Online Resource Supplementary Table 6).

The frequency of PET and PIH did not differ between the two randomisation groups (Table 2). Additionally, there were no differences between groups in the gestation at which anti-hypertensive agents were started (placebo median 37.5 weeks (IQR 36.1, 39.3), cholecalciferol median 37.4 weeks (IQR 35.1,40.4) *p* = 0.86), nor the gestation at delivery for women with PET (*n* = 26) (placebo median 39.4 weeks (IQR 38.0,40.4), cholecalciferol 37.9 weeks (IQR 36.1,40.3) *p* = 0.37).

**Table 2:** Incidence of PIH/PET by randomisation group, for all women and by parity

	Placebo	Cholecalciferol	p*
All women	*n* = 368	*n* = 366	
Pregnancy Induced Hypertension, *n* (%)	6 (1.6%)	13 (3.6%)	0.10
Preeclampsia, *n* (%)	12 (3.3%)	14 (3.8%)	0.68
Nulliparous women	*n* = 154	*n* = 147	
Pregnancy Induced Hypertension, *n* (%)	2 (1.3%)	9 (5.8%)	0.06
Preeclampsia, *n* (%)	8 (5.2%)	12 (7.7%)	0.49
Multiparous women	*n* = 210	*n* = 209	
Pregnancy Induced Hypertension, *n* (%)	4 (1.9%)	4 (1.9%)	0.99
Preeclampsia, *n* (%)	4 (1.9%)	2 (1.0%)	0.69

*p-value in the stratified analyses from Fisher’s exact test. Information on parity was not available for 4 women assigned to the placebo group and 10 women in the cholecalciferol group

## Discussion

In this large, randomised placebo-controlled trial of vitamin D supplementation in pregnancy, 1000 IU/day cholecalciferol from 14 to 17 weeks’ gestation until delivery did not reduce maternal systolic or diastolic BP during mid and late pregnancy nor the incidence of PIH or PET.

The effect of vitamin D supplementation on PET incidence has previously been explored in several intervention studies, although many are small and likely underpowered to detect an effect [14]. This is the largest RCT of antenatal vitamin D supplementation to assess this outcome. Whilst two recent meta-analyses have suggested that pregnancy vitamin D supplementation can reduce the risk of PET, care should be taken in the interpretation of these. Fogacci et al. excluded seventeen intervention studies in which no participant (in either the control group or supplementation group) developed PET from the pooled analysis, an approach which is likely to bias towards the intervention [14]. The more recent meta-analysis by AlSubai et al. also reported a protective effect of vitamin D supplementation on PET risk, but over half the weighting was applied to one study deemed to be at high risk of bias, in which treatment allocation was based on hospital attended and a complex algorithm stratified by maternal vitamin D status [13, 15]. Furthermore, in both these meta-analyses several of the included studies specifically recruited women deemed to be at higher risk of PET, resulting in high incidences of PET in the control groups compared to the background population. Additionally, there was marked variation in the supplementation protocols between studies.

Despite sufficient power in the MAVIDOS trial to detect a clinically relevant difference in PIH incidence, this was not found. Smaller studies have detected a difference in this outcome, but this could reflect the higher reported PIH/PET frequency in the control groups compared to our study. For example, Sablok et al.reported PIH/PET occurring in 21.1% and 11.1% of unsupplemented and supplemented groups, respectively, but neither blinding nor the definitions used for PIH/PET were reported [26]. Similarly, Ali et al., reported a reduction in PET incidence from 8.6% to 1.2% using 4000 IU/day compared to 400 IU/day [27]. For this reason, we investigated the effect of cholecalciferol supplementation on BP throughout pregnancy to determine whether more subtle differences in BP were evident.

Vitamin D is proposed to affect BP via regulation of endothelial function [28]. However, in contrast to studies in adults with metabolic syndrome and essential hypertension, in whom vitamin D supplementation reduced systolic and diastolic BP [29, 30], neither were lowered by supplementation in this study of pregnant women. Supplementation was commenced at 14–17 weeks; earlier use of supplementation may be required to effect placentation. Indeed, the aforementioned trial by Ali et al., which reported a positive effect, commenced supplementation at 6–12 weeks gestation [27].

Currently, women in the UK are advised to take 400 IU/day vitamin D supplementation during pregnancy to reduce the likelihood of vitamin D deficiency [31]. Although an effect of 1000 IU/day cholecalciferol on maternal BP was not demonstrated in this study, other benefits of this dose of vitamin D supplementation in pregnancy have been shown [20, 32–36], and therefore this finding should not alter current public health advice regarding vitamin D supplementation in pregnancy.

A strength of our study is the size of the study population; MAVIDOS is one of the largest RCTs of pregnancy vitamin D supplementation completed to date. However, this analysis has limitations, including the exclusion of women with very low levels (< 25 nmol/l) of 25(OH)D at recruitment due to ethical and governance issues. Very deficient women may benefit the most from vitamin D supplementation, potentially leading to the lack of an observed effect on BP. Over 95% of the MAVIDOS participants were of White ethnicity, which reflects the local population of recruited participants, but limits the generalisability of the study findings. For example, individuals from Afro-Caribbean backgrounds are recognised to be at higher risk of both hypertensive disorders of pregnancy and vitamin D deficiency [37]. The reliance on non-standardised clinical measurements of BP is a weakness, as different methods and equipment may have been used for measurements. Additionally, BP measurements were not available for all participants at all gestational windows. Women at higher risk of raised BP or PIH/PET may possibly have had more frequent monitoring, although this would have been expected to be similar for both randomisation groups.

These post-hoc analyses are hypothesis-generating, rather than part of the pre-specified analysis plan for MAVIDOS [17]. However, the MAVIDOS trial provides an important opportunity to assess the effects of vitamin D supplementation in pregnancy on other outcomes. In this analysis, we have combined real-world clinical data collecting during clinical care into an RCT enabling additional outcomes to be evaluated without the high costs of a new RCT. An analysis plan was written and published prior to the analysis being performed to increase the transparency of our work [16, 19].

## Conclusion

In this post-hoc analysis of the MAVIDOS randomised placebo-controlled trial, 1000 IU/day vitamin D supplementation in pregnancy did not lower maternal BP assessed at multiple gestations from mid-pregnancy and did not reduce the incidence of PIH or PET.

## Supplementary Information

Below is the link to the electronic supplementary material.

Supplementary file1 (DOCX 31 KB)
