# Supplementary material for: The effect of pregnancy vitamin D supplementation on maternal blood pressure: real-world data analysis within the MAVIDOS randomised placebo-controlled trial
Source: Arch Gynecol Obstet. 2025 Jan 30;311(4):941–9. doi: 10.1007/s00404-025-07958-z (PMC11985589; doi:10.1007/s00404-025-07958-z)
Supplement: Supplementary file 1 — Supplementary file1 (DOCX 31 KB) [file 404_2025_7958_MOESM1_ESM.docx]

**The effect of pregnancy vitamin D supplementation on maternal blood pressure: a real-world data analysis within the MAVIDOS randomized placebo-controlled trial.**

**Natasha L Citeroni-Clark, Stefania D’Angelo, Sarah R Crozier, Alexandra Kermack, Keith M Godfrey, Cyrus Cooper, Nicholas C Harvey, Rebecca J Moon**

**SUPPLEMENTARY TABLES**

**Supplementary Table 1.** Baseline characteristics of women included in this analysis in comparison to those that participated in the MAVIDOS study but were not included in this analysis

|  | Not included (n=231) | | Included in the analysis (n=734) | |
| --- | --- | --- | --- | --- |
|  | n |  | n |  |
| Smoking at recruitment, n(%) | 198 | 12 (6.1%) | 731 | 60 (8.2%) |
| White ethnicity, n(%) | 198 | 184 (92.9%) | 730 | 696 (95.3%) |
| Nulliparous, n(%) | 197 | 92 (46.7%) | 729 | 310 (42.5%) |
| Educated to A levels or higher, n(%) | 198 | 166 (83.8%) | 727 | 551 (75.8%) |
| 25(OH)D in early pregnancy (nmol/l), mean (SD) | 216 | 50.4 (18.9) | 717 | 45.0 (16.4) |
| Age at delivery (years), mean (SD) | 198 | 31.1 (5.3) | 734 | 31.2 (5.1) |

**Supplementary Table 2.** Baseline characteristics for nulliparous women

|  | **Placebo (n=154)** | | **Cholecalciferol (n=156)** | |
| --- | --- | --- | --- | --- |
|  | **n** |  | **n** |  |
| Age at delivery (years), mean (SD) | 154 | 30.2 (5.3) | 156 | 29.9 (5.1) |
| White ethnicity, n (%) | 154 | 148 (96.1%) | 155 | 148 (95.5%) |
| Height (cm), mean (SD) | 153 | 166.2 (6.3) | 156 | 166.1 (6.3) |
| Weight (kg), mean (SD) | 154 | 71.8 (13.2) | 156 | 69.4 (14.4) |
| BMI (kg/m^2^), mean (SD) | 153 | 26.1 (4.7) | 156 | 25.1 (4.7) |
| Smoking in early pregnancy, n (%) | 154 | 12 (7.8%) | 156 | 10 (6.4%) |
| Moderate/strenuous physical activity (hours/week), n (%) | 121 | 0.98 (0.90) | 125 | 0.95 (0.80) |
| Educated to A level or more, n (%) | 151 | 122 (80.8%) | 156 | 131 (84.0%) |
| Pre-existing hypertension, n (%) | 154 | 2 (1.3%) | 156 | 1 (0.6%) |
| Systolic BP at booking (mmHg), mean (SD) | 132 | 110.0 (10.8) | 130 | 109.0 (10.3) |
| Diastolic BP at booking (mmHg), mean (SD) | 133 | 66.0 (8.7) | 130 | 65.4 (7.6) |
| 25(OH)D at randomisation (nmol/l), mean (SD) | 150 | 45.2 (16.0) | 154 | 47.5 (15.8) |

**Supplementary Table 3.** Baseline characteristics for multiparous women

|  | **Placebo (n=210)** | | **Cholecalciferol (n=209)** | |
| --- | --- | --- | --- | --- |
|  | **n** |  | **n** |  |
| Age at delivery (years), mean (SD) | 210 | 32.0 (4.9) | 209 | 32.1 (4.9) |
| White ethnicity, n (%) | 210 | 198 (94.3%) | 207 | 198 (95.7%) |
| Height (cm), mean (SD) | 207 | 166.0 (6.9) | 208 | 165.0 (6.2) |
| Weight (kg), mean (SD) | 210 | 74.1 (14.5) | 209 | 72.0 (13.8) |
| BMI (kg/m^2^), mean (SD) | 207 | 26.9 (5.1) | 208 | 26.5 (5.0) |
| Smoking in early pregnancy, n (%) | 209 | 20 (9.6%) | 208 | 18 (8.7%) |
| Moderate/strenuous physical activity (hours/week), mean (SD) | 162 | 0.93 (0.74) | 161 | 0.83 (0.54) |
| Educated to A level or more, n (%) | 209 | 147 (70.3%) | 207 | 148 (71.5%) |
| Pre-existing hypertension, n (%) | 210 | 2 (1.0%) | 209 | 2 (1.0%) |
| Systolic BP at booking (mmHg), mean (SD) | 176 | 109.6 (11.0) | 174 | 109.2 (10.5) |
| Diastolic BP at booking (mmHg), mean (SD) | 176 | 65.5 (8.7) | 174 | 65.8 (8.7) |
| 25(OH)D at randomisation (nmol/l), mean (SD) | 206 | 44.0 (16.8) | 202 | 44.3 (16.7) |

**Supplementary Table 4:** Blood pressure during pregnancy by randomisation to placebo or 1000 IU/day cholecalciferol from 14-17 weeks’ gestation until delivery in nulliparous women

|  | **Systolic (mmHg)** | | | | | **Diastolic (mmHg)** | | | | |
| --- | --- | --- | --- | --- | --- | --- | --- | --- | --- | --- |
| **Gestation of BP assessment (weeks^+days^)** | **Placebo** | | **Cholecalciferol** | | **p** | **Placebo** | | **Cholecalciferol** | | **p** |
|  | **N** | **Mean (SD)** | **N** | **Mean (SD)** |  | **N** | **Mean (SD)** | **N** | **Mean (SD)** |  |
| <12^+6^ | 132 | 110 (11) | 130 | 109 (10) | 0.44 | 133 | 66 (9) | 130 | 65 (8) | 0.57 |
| 23^+0^-24^+6^ | 63 | 109 (12) | 62 | 108 (10) | 0.66 | 63 | 65 (8) | 62 | 64 (7) | 0.53 |
| 27^+0^-28^+6^ | 106 | 110 (11) | 111 | 111 (11) | 0.94 | 105 | 66 (8) | 111 | 65 (8) | 0.64 |
| 33^+0^-35^+6^ | 142 | 112 (11) | 144 | 113 (11) | 0.93 | 143 | 68 (8) | 144 | 67 (8) | 0.40 |
| 37^+0^-38^+6^ | 132 | 115 (11) | 123 | 115 (13) | 0.99 | 132 | 71 (9) | 123 | 71 (9) | 0.94 |
| 39^+0^-40^+6^ | 90 | 118 (14) | 97 | 119 (12) | 0.37 | 90 | 72 (10) | 97 | 74 (9) | 0.17 |
| ≥41^+0^ | 26 | 121 (11) | 20 | 125 (14) | 0.32 | 26 | 73 (11) | 20 | 75 (15) | 0.51 |

**Supplementary Table 5.** Blood pressure during pregnancy by randomisation to placebo or 1000 IU/day cholecalciferol from 14-17 weeks’ gestation until delivery in multiparous women

|  | **Systolic (mmHg)** | | | | | **Diastolic (mmHg)** | | | | |
| --- | --- | --- | --- | --- | --- | --- | --- | --- | --- | --- |
| **Gestation of BP assessment (weeks^+days^)** | **Placebo** | | **Cholecalciferol** | | **p** | **Placebo** | | **Cholecalciferol** | | **p** |
|  | **N** | **Mean (SD)** | **N** | **Mean (SD)** |  | **N** | **Mean (SD)** | **N** | **Mean (SD)** |  |
| <12^+6^ | 176 | 110 (11) | 174 | 109 (11) | 0.72 | 176 | 66 (9) | 174 | 66 (9) | 0.72 |
| 23^+0^-24^+6^ | 60 | 109 (11) | 56 | 110 (11) | 0.73 | 61 | 65 (8) | 56 | 65 (8) | 0.78 |
| 27^+0^-28^+6^ | 148 | 109 (11) | 149 | 109 (11) | 0.84 | 148 | 64 (7) | 149 | 65 (7) | 0.20 |
| 33^+0^-35^+6^ | 188 | 111 (11) | 180 | 110 (11) | 0.28 | 188 | 66 (8) | 183 | 65 (8) | 0.58 |
| 37^+0^-38^+6^ | 174 | 113 (11) | 174 | 111 (12) | 0.19 | 175 | 68 (9) | 174 | 68 (8) | 0.83 |
| 39^+0^-40^+6^ | 126 | 114 (12) | 110 | 112 (11) | 0.17 | 127 | 70 (9) | 110 | 68 (7) | 0.05 |
| ≥41^+0^ | 24 | 113 (10) | 22 | 112 (11) | 0.80 | 24 | 72 (10) | 22 | 67 (7) | 0.10 |

**Supplementary Table 6.** Blood pressure in pregnancy in all women randomised to either placebo or 1000 IU/day cholecalciferol from 14-17 weeks gestation until delivery

|  | **Systolic (mmHg)** | | | | | **Diastolic (mmHg)** | | | | |
| --- | --- | --- | --- | --- | --- | --- | --- | --- | --- | --- |
| **Gestation of BP assessment (weeks^+days^)** | **Placebo** | | **Cholecalciferol** | | **p** | **Placebo** | | **Cholecalciferol** | | **p** |
|  | **N** | **Mean (SD)** | **N** | **Mean (SD)** |  | **N** | **Mean (SD)** | **N** | **Mean (SD)** |  |
| <12^+6^ | 310 | 110 (11) | 305 | 109 (10) | 0.41 | 311 | 66 (9) | 305 | 66 (8) | 0.86 |
| 23^+0^-24^+6^ | 126 | 110 (12) | 118 | 109 (11) | 0.82 | 127 | 65 (8) | 118 | 65 (8) | 0.82 |
| 27^+0^-28^+6^ | 258 | 110 (11) | 261 | 110 (11) | 0.88 | 257 | 65 (8) | 261 | 65 (7) | 0.68 |
| 33^+0^-35^+6^ | 333 | 112 (11) | 324 | 111 (11) | 0.43 | 334 | 67 (8) | 327 | 66 (8) | 0.31 |
| 37^+0^-38^+6^ | 308 | 114 (11) | 298 | 113 (12) | 0.26 | 309 | 69 (9) | 298 | 69 (9) | 0.74 |
| 39^+0^-40^+6^ | 217 | 115 (13) | 207 | 115 (12) | 0.96 | 218 | 71 (10) | 207 | 71 (9) | 0.94 |
| ≥41^+0^ | 50 | 117 (11) | 42 | 118 (14) | 0.72 | 50 | 72 (10) | 42 | 71 (12) | 0.64 |
